# Supplementary material for: Fine scale human mobility changes within 26 US cities in 2020 in response to the COVID-19 pandemic were associated with distance and income
Source: PLOS Glob Public Health. 2023 Jul 21;3(7):e0002151. doi: 10.1371/journal.pgph.0002151 (PMC10361529; doi:10.1371/journal.pgph.0002151)

<u>S1 Text. Model results with alternative distance quartiles</u>

The results in the main manuscript use distance quartiles based on trips over the whole study period. However, since the frequency of trips of different distances changed different amounts in response to the COVID-19 pandemic, here we present the results of these models when distance quartiles are based on only trips taken in February 2020 (which we took as the baseline period for all analyses). The thresholds for each quartile changed very little and therefore the modelled results were also very similar.

S1 Text Fig 1: Effects of distance, income, relative case rates, and age on rate of decrease in travel between February 1- April 3 when using distance thresholds defined using trips only during February.

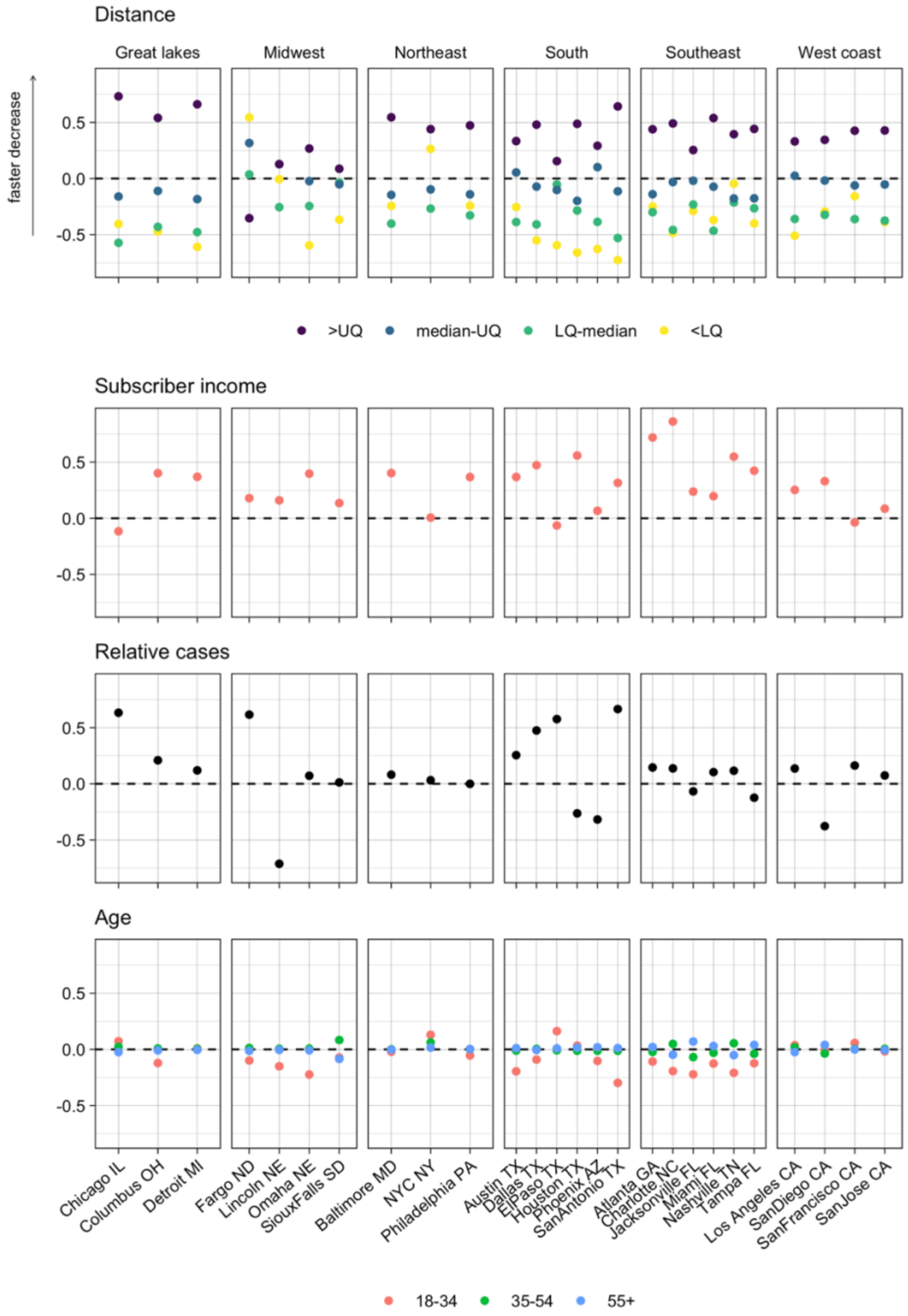

S1 Text Fig 2: Effects of distance, income and age on trip rates between June 1 – August 31 compared to baseline travel when using distance thresholds defined using trips only during February.

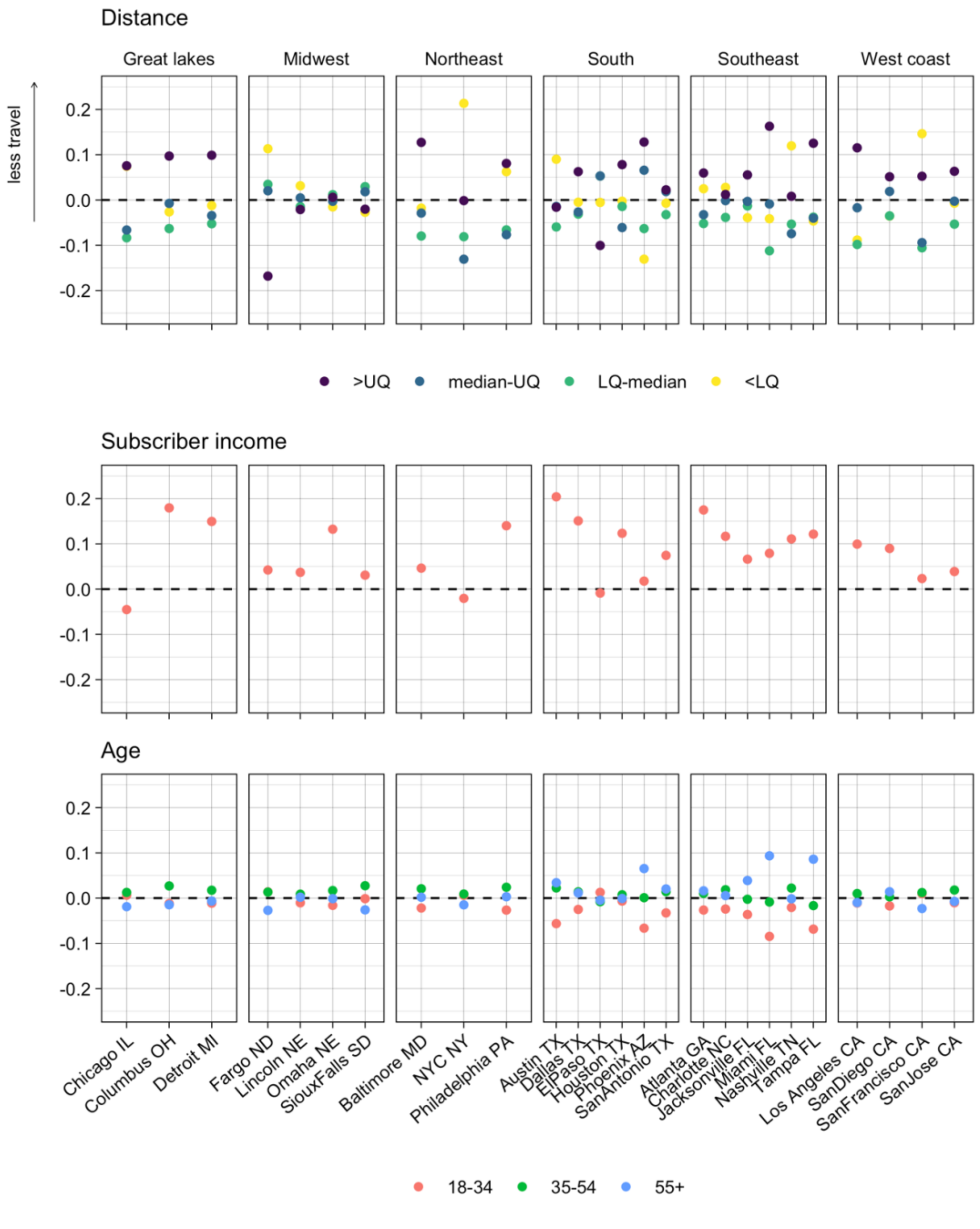

Supplement: S1 Text — (PDF) [file pgph.0002151.s003.pdf]
