# Supplementary material for: Fine scale human mobility changes within 26 US cities in 2020 in response to the COVID-19 pandemic were associated with distance and income
Source: PLOS Glob Public Health. 2023 Jul 21;3(7):e0002151. doi: 10.1371/journal.pgph.0002151 (PMC10361529; doi:10.1371/journal.pgph.0002151)

S2 Text. Demographic distributions

The distributions of individual income for the subscribers in the dataset in each city is shown in S2 Text Fig 1 and for the general population of the US by region from Census data in S2 Text Fig 2. A clear overrepresentation of higher income groups can be seen in all cities compared to the general population in any region.

S2 Text Fig 1: Proportions of subscribers in each city in each income bracket

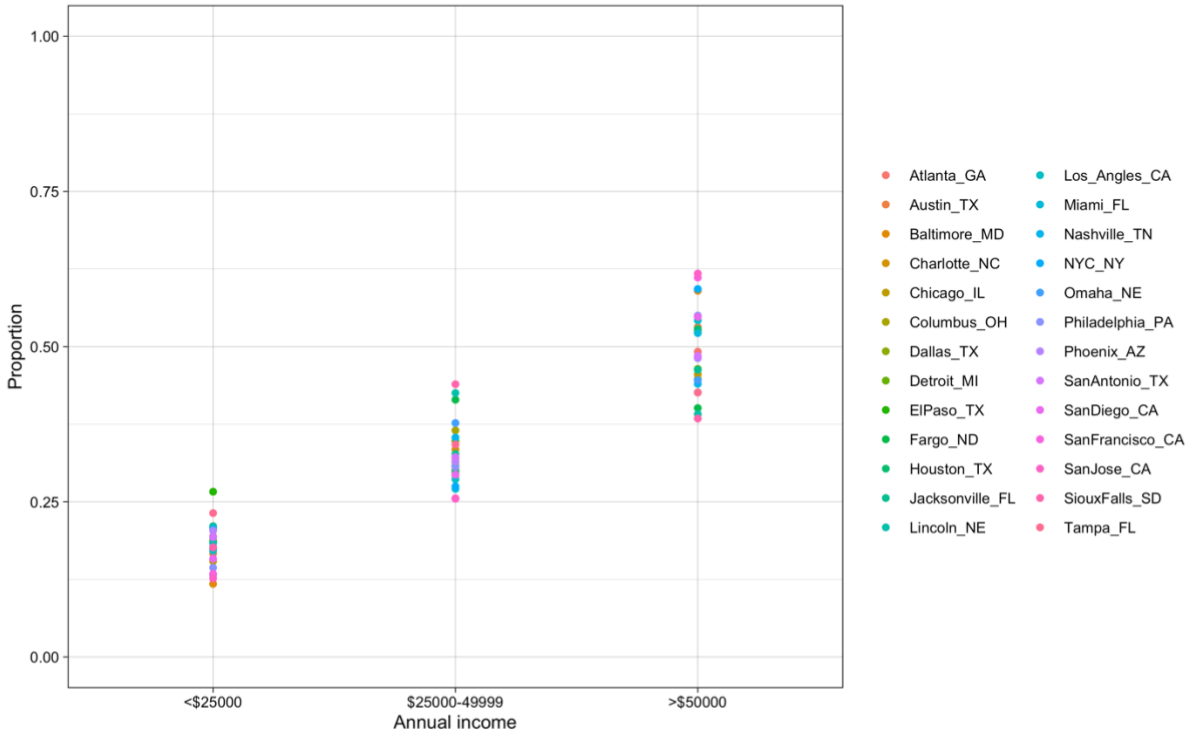

S2 Text Fig 2: Proportion of general population in regions of the US in each income bracket [60]

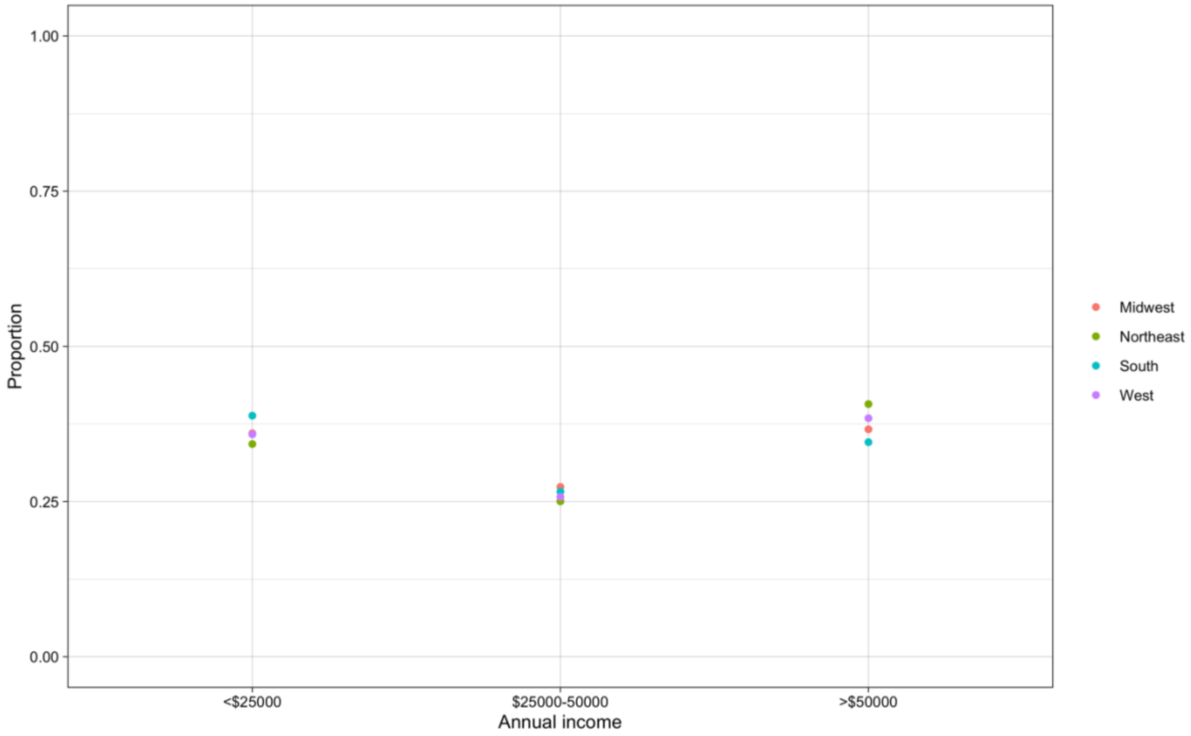

The distributions of age for the subscribers in the dataset in each city is shown in S2 Text Fig 3 and for the general population of the US from Census data in S2 Text Fig 4. Here the older age groups are overrepresented in the subscriber data compared to the general population, which is clear despite the slight mismatch in the age ranges of the youngest group.

S2 Text Fig 3: Proportions of subscribers in each city in each age bracket

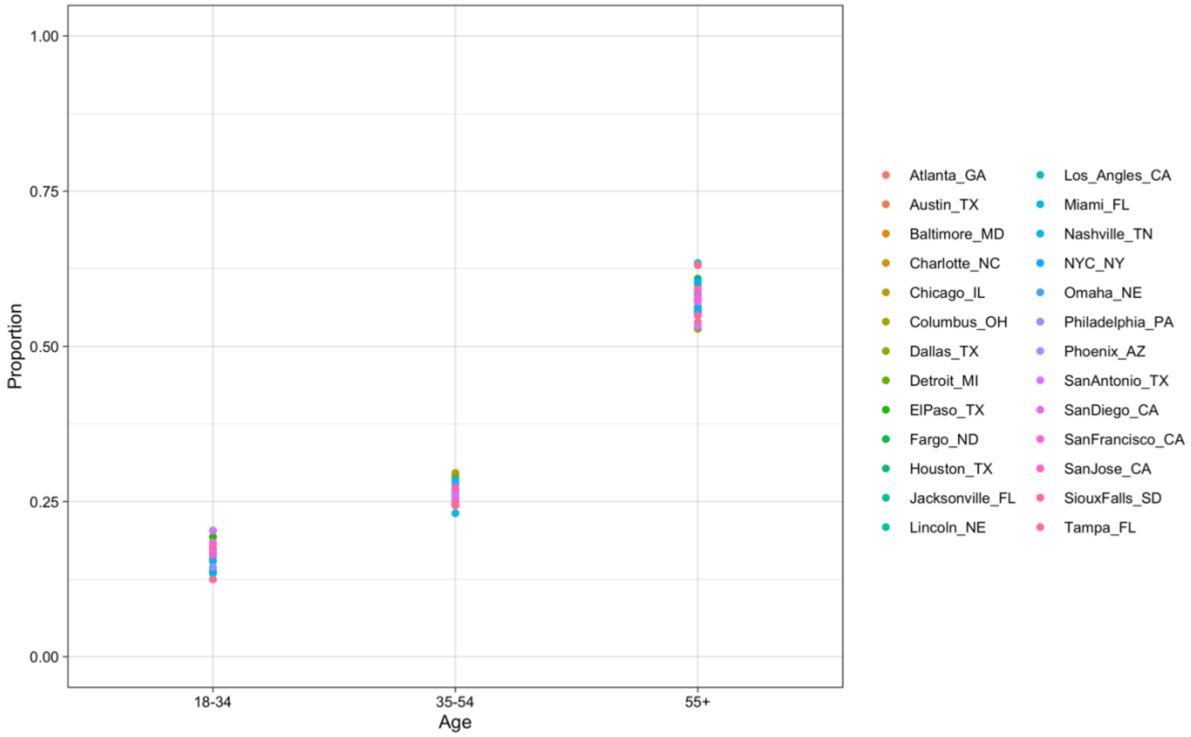

S2 Text Fig 4: Proportion of general US population in each age bracket [61]

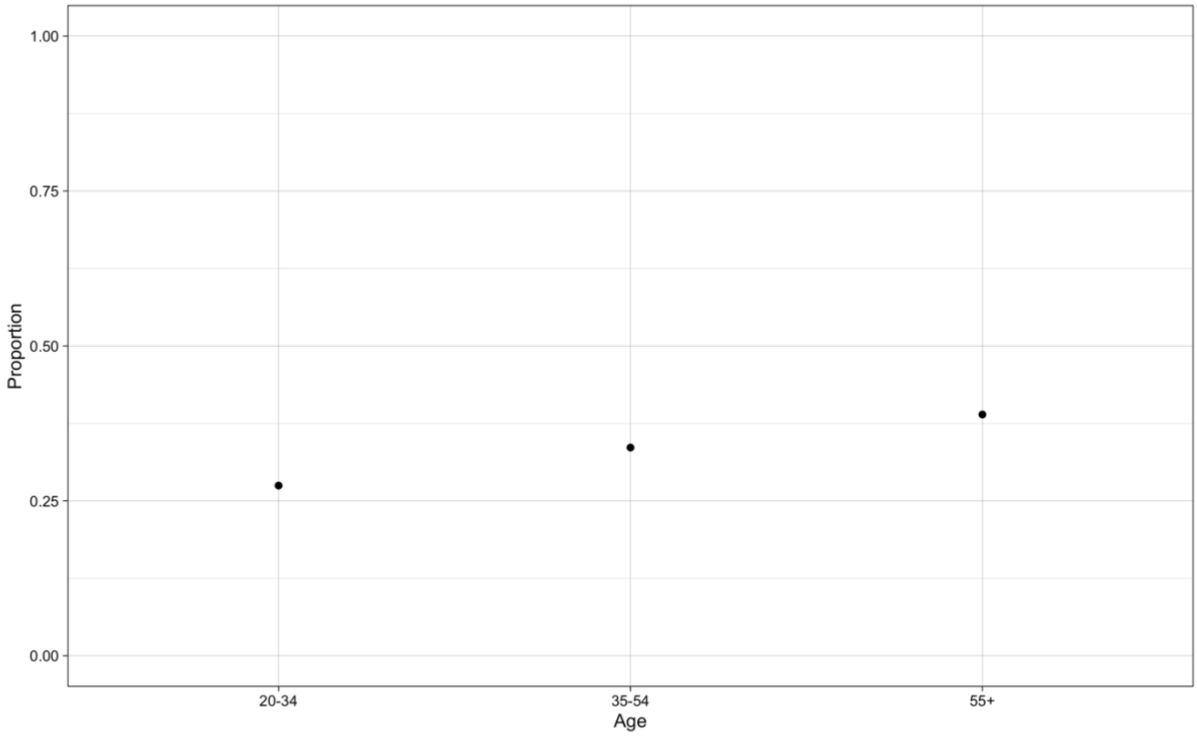

Supplement: S2 Text — (PDF) [file pgph.0002151.s004.pdf]
