# Supplementary material for: Fine scale human mobility changes within 26 US cities in 2020 in response to the COVID-19 pandemic were associated with distance and income
Source: PLOS Glob Public Health. 2023 Jul 21;3(7):e0002151. doi: 10.1371/journal.pgph.0002151 (PMC10361529; doi:10.1371/journal.pgph.0002151)
Supplement: S3 Text — (PDF) [file pgph.0002151.s005.pdf]

S3 Text. Prior distributions

Modelling initial decrease in mobility

All effect sizes were given Normal priors centred at zero with standard deviations of 1. Baseline trip rates were parameterised on the log scale with Normal priors centred at -3 with standard deviations of 0.5. Note that baseline trip rates were treated as random parameters and integrated out (via a Laplace approximation) at each step of the optimisation. The weekly city-wide rates of decrease in mobility, c_k, were also parameterised on the log scale with an AR(1) prior, with $\phi = 1,\ c = 0, \epsilon = 0.1$.

Modelling mobility over summer

All effect sizes were given Normal priors centred at zero with standard deviations of 1.
