# Supplementary material for: Fine scale human mobility changes within 26 US cities in 2020 in response to the COVID-19 pandemic were associated with distance and income
Source: PLOS Glob Public Health. 2023 Jul 21;3(7):e0002151. doi: 10.1371/journal.pgph.0002151 (PMC10361529; doi:10.1371/journal.pgph.0002151)
Supplement: S4 Text — (PDF) [file pgph.0002151.s006.pdf]

S4 Text. Modification to Poisson likelihood for censored observations

First, consider the model of decreasing mobility between February 2 – April 4. The number of trips between zip codes i and j (in either direction) in age group a at time t is denoted Y_{ijat}. For simplicity we can considered a fixed time and age group and just write Y_{ij} for this variable. This is the sum of trips from i to j and j to i, which we denote Y_{i-j} and Y_{j-i} respectively.

Let λ be the mean of the Poisson process implied by some parameter values (again just for notational convenience), so the likelihood when Y_{ij} is known exactly is

$$Y_{ij} \sim Poisson(\lambda)$$

or in other words

$$p(Y_{ij}|\lambda)$$

where p is the probability mass function of a Poisson distribution with mean λ.

There are two possibilities for when the total Y_{ij} isn't known exactly, either one of Y_{i-j} and Y_{j-i} are below 50 (but not both) or both are below 50. Suppose just one is below 50 and let it be Y_{i-j} be known (otherwise switch the indices). The total Y_{ij} could therefore be any value between Y_{i-j} and $Y_{i-j} + 49$ and so the likelihood is

$$p\left(Y_{i-j} \leq Y_{ij} \leq Y_{i-j} + 49 \middle| \lambda\right) = \sum_{k=0}^{49} p(Y_{i-j} + k|\lambda)$$

Similarly, if both are below 50 then Y_{ij} could take any value between 0 and 99, yielding a likelihood

$$p\left(0 \leq Y_{ij} \leq 99 \middle| \lambda\right) = \sum_{k=0}^{99} p(k|\lambda)$$

Likelihoods were modified in a similar way for the model of mobility between June 1 – August 31, where the uncertainty (and therefore the number of values summed over) accumulates for each week that is censored.
