# Supplementary material for: Fine scale human mobility changes within 26 US cities in 2020 in response to the COVID-19 pandemic were associated with distance and income
Source: PLOS Glob Public Health. 2023 Jul 21;3(7):e0002151. doi: 10.1371/journal.pgph.0002151 (PMC10361529; doi:10.1371/journal.pgph.0002151)
Supplement: S5 Text — (PDF) [file pgph.0002151.s007.pdf]

S5 Text. Comparison to gravity model

We compare the performance of the original model and where a gravity model was used to model baseline travel. Here we used the Bayesian information criterion (BIC) [46, 47], which is defined as

$$BIC = k\log(n) - 2\log(\hat{L})$$

where k is the number of parameters in the model, n is the number of data points, and L is the maximum likelihood value. Similarly to the Akaike information criterion (AIC), the BIC evaluates model performance based on the likelihood while penalising the number of parameters in the model. This penalisation term is greater in BIC compared to AIC, and therefore we would expect BIC to be more favourable towards the gravity model given the large number of parameters used in the original model. Despite this, BIC values were consistently smaller for the original model compared to the gravity model, suggested the original model performs better.

City	Original model BIC	Gravity model BIC
Atlanta	3840161.2	258224101
Austin	1222027.15	64803466.9
Baltimore	5087018.43	261635336
Charlotte	2067629.99	173940690
Chicago	7706554.13	458064822
Columbus	1938871.43	102160615
Dallas	4518151.76	137857475
Detroit	5297367.31	293064915
El Paso	1195483.35	49771459.6
Fargo	232714.497	11924885.5
Houston	4371970.12	245544784
Jacksonville	719335.198	45020737.1
Lincoln	575325.226	74694180.3
Los Angeles	12295065.1	700370925
Miami	2744751.23	159705789
Nashville	1627853.07	591536474
NYC	43231266.4	1235370300
Omaha	1257801.97	333591669
Philadelphia	6469144.31	271283519
Phoenix	3949295.59	1222806194
San Antonio	1306702.55	55910501.3
San Diego	3323471.85	217148993
San Francisco	5093222.29	259613186
San Jose	3227666.01	648414497
Sioux Falls	257991.936	73655260.1
Tampa	3013279.97	175615714
