# Supplementary material for: Fine scale human mobility changes within 26 US cities in 2020 in response to the COVID-19 pandemic were associated with distance and income
Source: PLOS Glob Public Health. 2023 Jul 21;3(7):e0002151. doi: 10.1371/journal.pgph.0002151 (PMC10361529; doi:10.1371/journal.pgph.0002151)
Supplement: S1 Table — (PDF) [file pgph.0002151.s008.pdf]

S1 Table: Summary of all models used

	Model 1: Rate of decrease in mobility	Model 2: Summer mobility compared to baseline	Model 3: Comparison to model 1 – rate of decrease in mobility
Time frame	February 1 – April 4	June 1 – August 31	February 1 – April 4
Baseline component	Baseline rate of travel between each pair of zips is a parameter to be learned.	Baseline rate of travel between each pair of zips is fixed at the rate learned in model 1.	Gravity model where propensity to travel and effect of distance, origin population and destination population on baseline travel is learned.
Changing mobility component	For each week there is a city-wide parameter for average rate of decrease of mobility. The effect of each explanatory variable on this city-wide average is learned.	Parameter β_0 describes average change in mobility rates between baseline and summer. The effect of each explanatory variable on this city-wide average is learned.	Same as model 1
Explanatory variables	Distance, proportion of high-income subscribers, case rate relative to city, age, median household income	Distance, proportion of high-income subscribers, age, median household income	Same as model 1
