# Supplementary material for: Fine scale human mobility changes within 26 US cities in 2020 in response to the COVID-19 pandemic were associated with distance and income
Source: PLOS Glob Public Health. 2023 Jul 21;3(7):e0002151. doi: 10.1371/journal.pgph.0002151 (PMC10361529; doi:10.1371/journal.pgph.0002151)
Supplement: S2 Table — (PDF) [file pgph.0002151.s009.pdf]

S2 Table. Proportion of subscribers in each city

City	% subscribers in city out of all subscribers in analysis
Atlanta GA	4.35
Austin TX	1.18
Baltimore MD	5.19
Charlotte NC	3.2
Chicago IL	6.27
Columbus OH	2.43
Dallas TX	3.07
Detroit MI	4.76
El Paso TX	0.83
Fargo ND	0.48
Houston TX	4.34
Jacksonville FL	1.17
Lincoln NE	0.72
Los Angeles CA	9.69
Miami FL	2.38
Nashville TN	2.54
NYC NY	13.31
Omaha NE	1.39
Philadelphia PA	5.47
Phoenix AZ	5.77
San Antonio TX	0.97
San Diego CA	2.81
San Francisco CA	3.87
San Jose CA	3.63
Sioux Falls SD	0.58
Tampa FL	3.36
