# Supplementary figures and images for: Fine scale human mobility changes within 26 US cities in 2020 in response to the COVID-19 pandemic were associated with distance and income
Source: PLOS Glob Public Health. 2023 Jul 21;3(7):e0002151. doi: 10.1371/journal.pgph.0002151 (PMC10361529; doi:10.1371/journal.pgph.0002151)

S1 Fig: Trips relative to baseline by age group in each city (a) – (e)

(a)

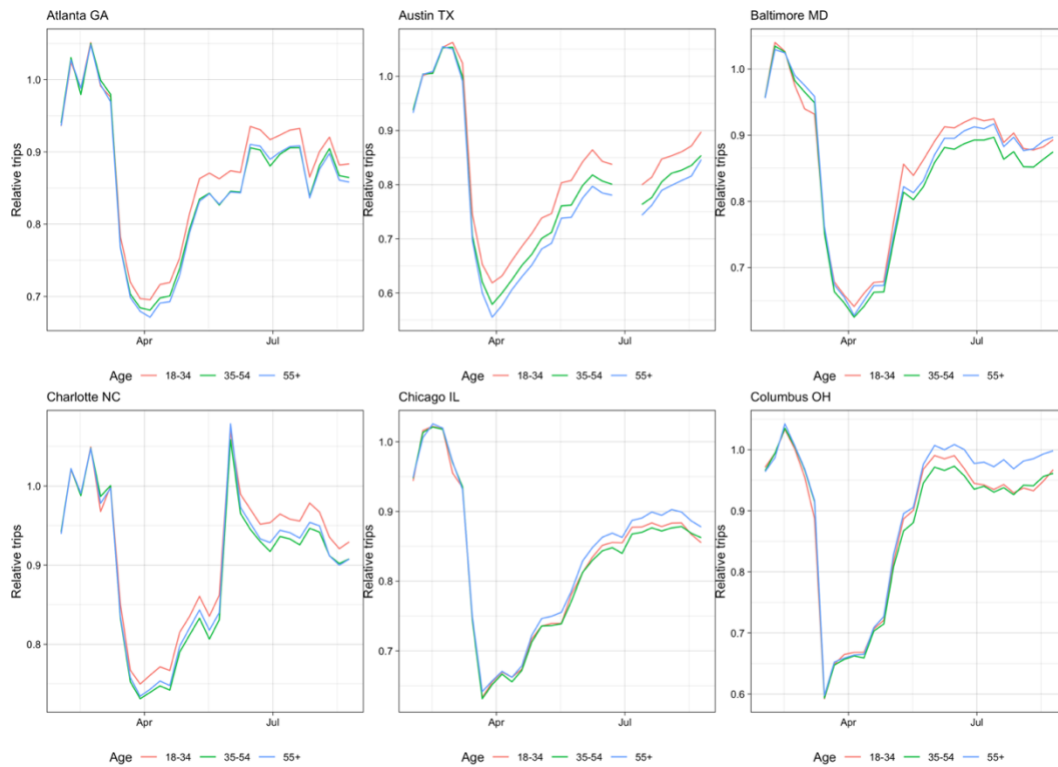

(b)

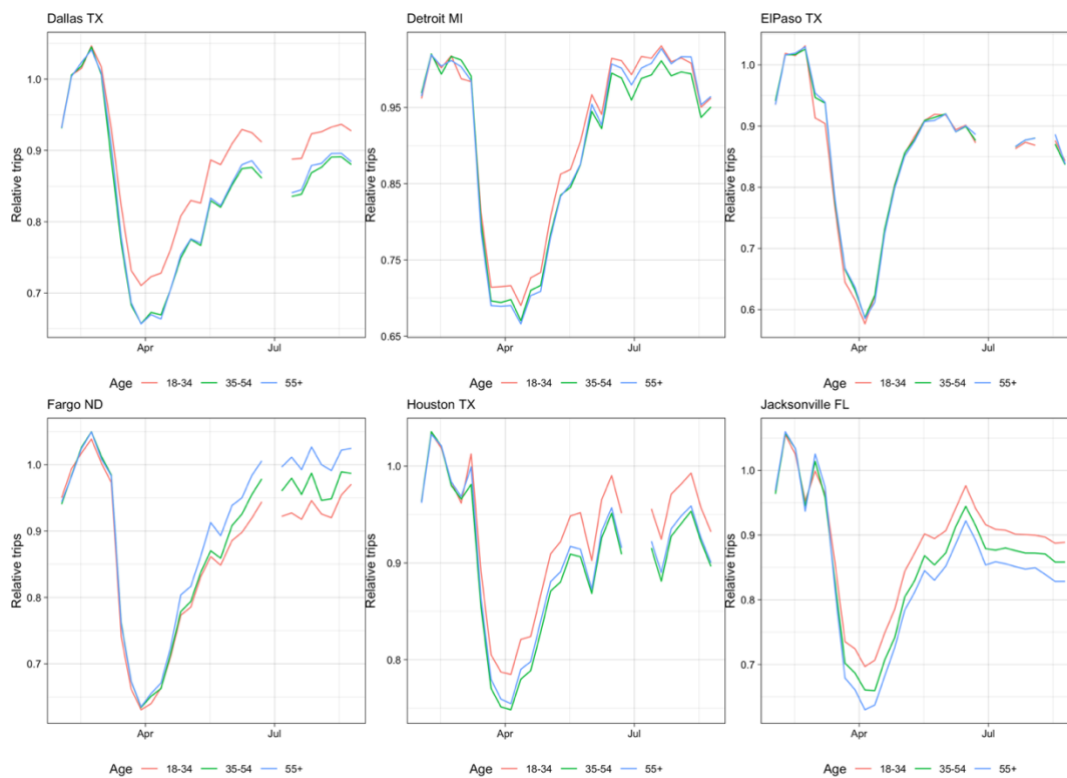

(c)

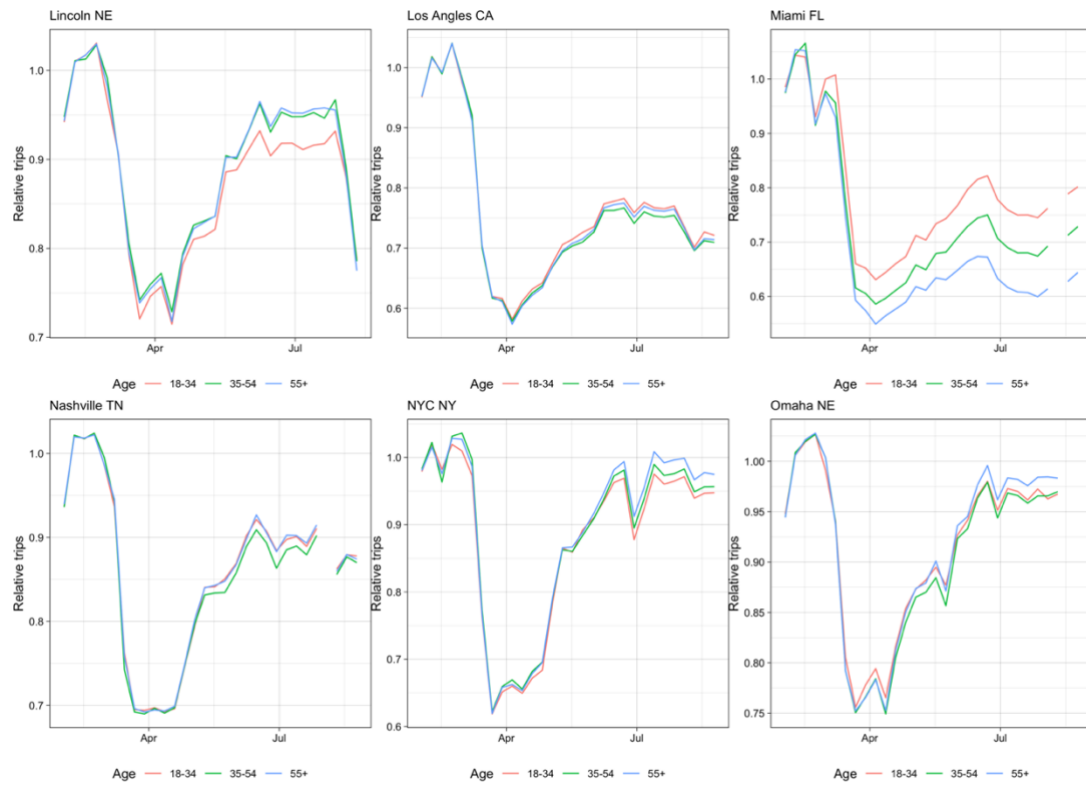

(d)

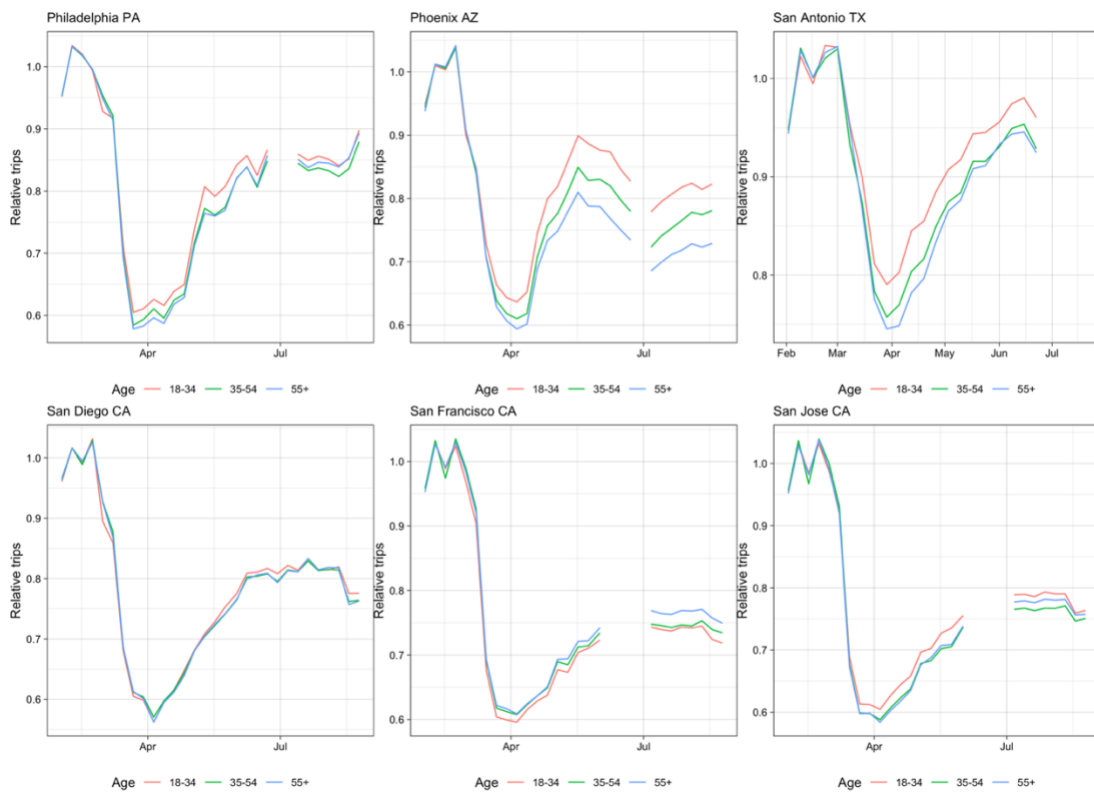

(e)

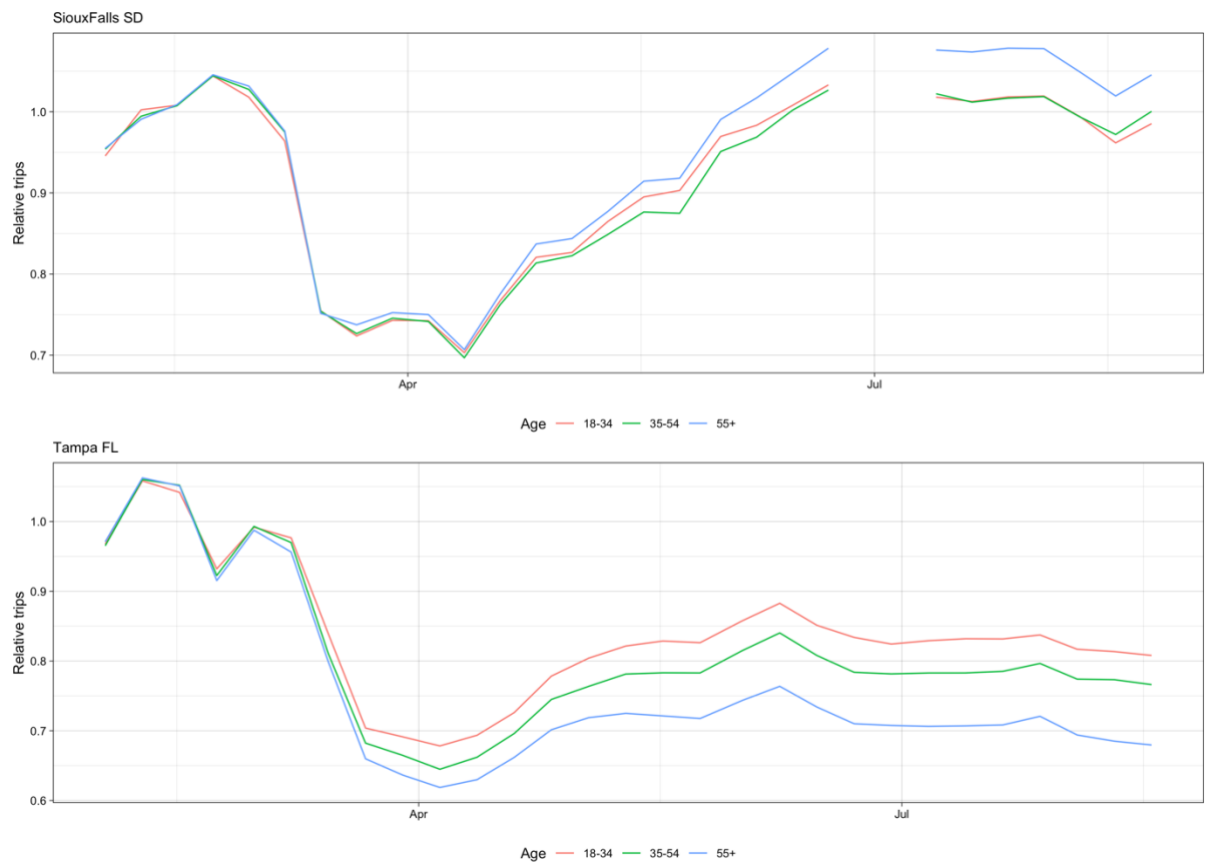

Supplement: S1 Fig — (PDF) [file pgph.0002151.s010.pdf]
