# Supplementary material for: Fine scale human mobility changes within 26 US cities in 2020 in response to the COVID-19 pandemic were associated with distance and income
Source: PLOS Glob Public Health. 2023 Jul 21;3(7):e0002151. doi: 10.1371/journal.pgph.0002151 (PMC10361529; doi:10.1371/journal.pgph.0002151)
Supplement: S2 Fig — (PDF) [file pgph.0002151.s011.pdf]

S2 Fig: Relative trips by distance quartile for each city (not including those presenting in the main text) (a) – (d)

(a)

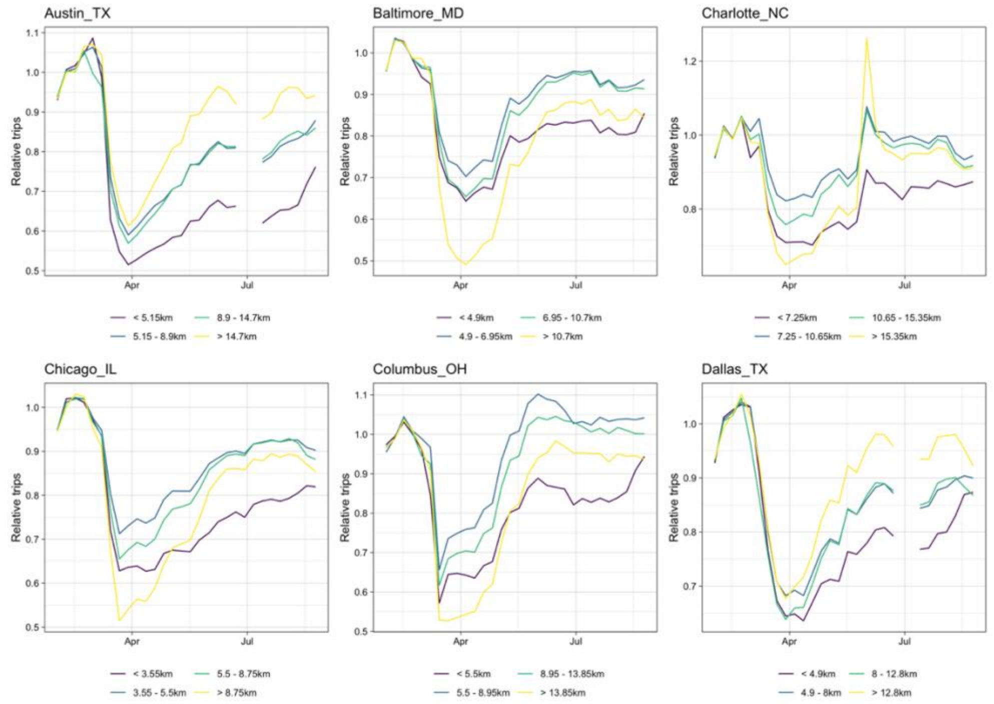

(b)

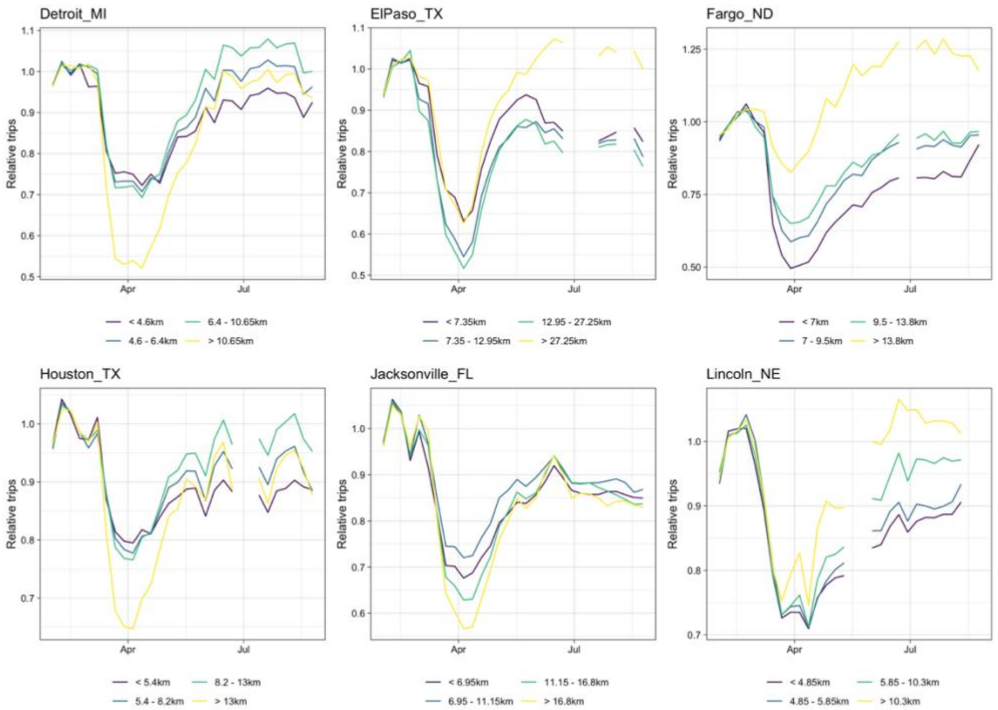

(c)

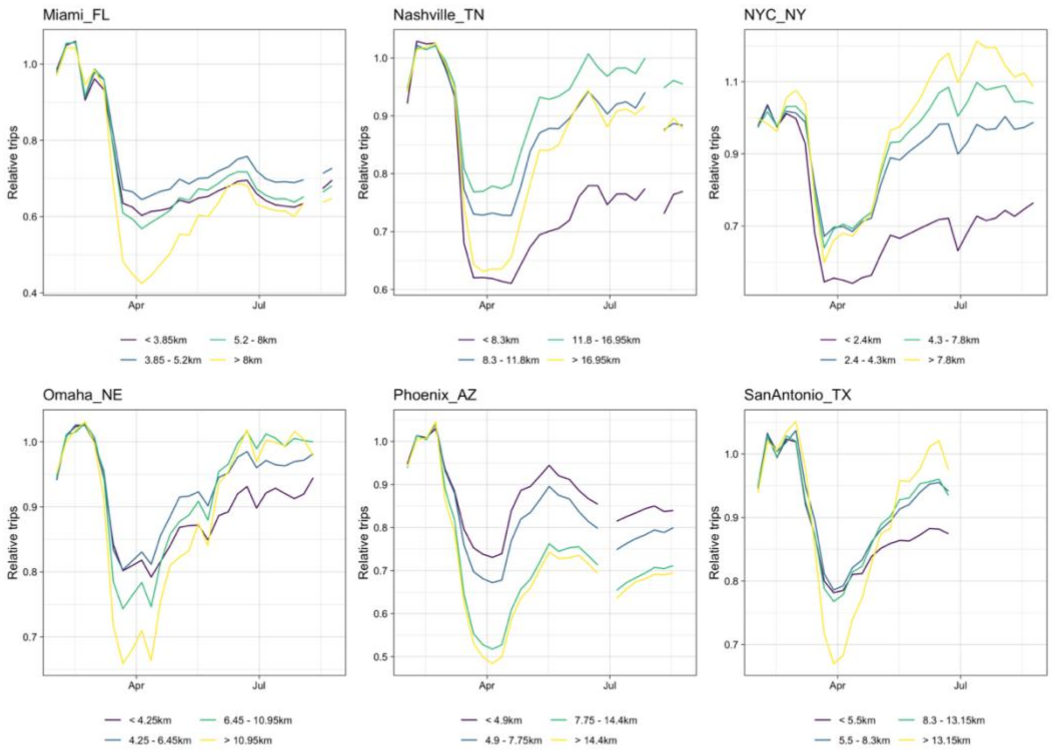
Miami_FL
Relative trips
Apr
Jul
< 3.85km
3.85 - 5.2km
5.2 - 8km
> 8km
Nashville_TN
< 8.3km
8.3 - 11.8km
11.8 - 16.95km
> 16.95km
NYC_NY
< 2.4km
2.4 - 4.3km
4.3 - 7.8km
> 7.8km
Omaha_NE
< 4.25km
4.25 - 6.45km
6.45 - 10.95km
> 10.95km
Phoenix_AZ
< 4.9km
4.9 - 7.75km
7.75 - 14.4km
> 14.4km
SanAntonio_TX
< 5.5km
5.5 - 8.3km
8.3 - 13.15km
> 13.15km

(d)

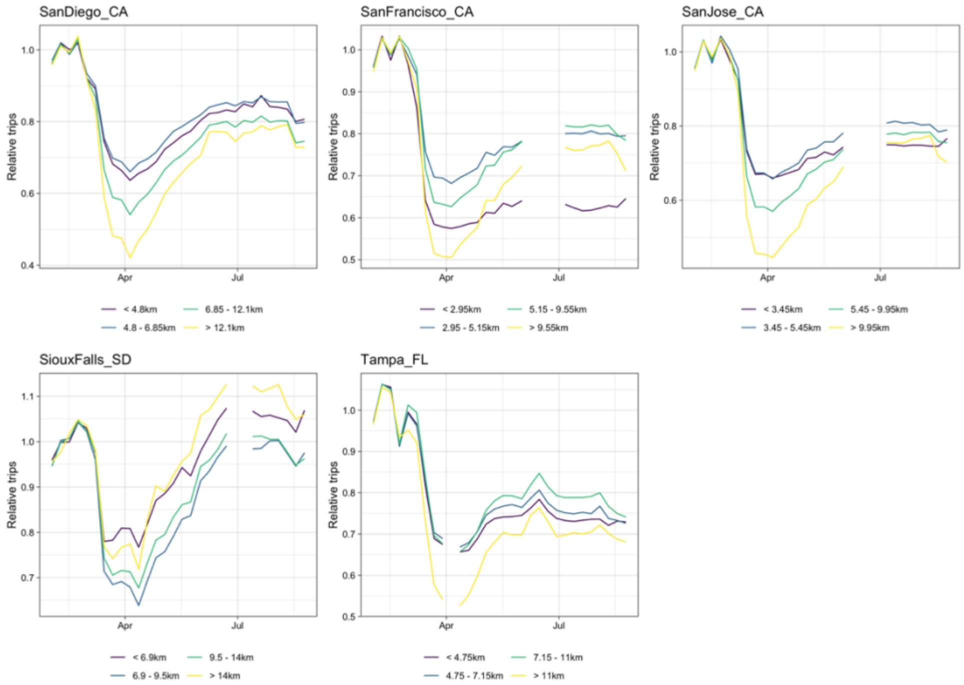
SanDiego_CA
Relative trips
Apr
Jul
< 4.8km
4.8 - 6.85km
6.85 - 12.1km
> 12.1km
SanFrancisco_CA
< 2.95km
2.95 - 5.15km
5.15 - 9.55km
> 9.55km
SanJose_CA
< 3.45km
3.45 - 5.45km
5.45 - 9.95km
> 9.95km
SiouxFalls_SD
< 6.9km
6.9 - 9.5km
9.5 - 14km
> 14km
Tampa_FL
< 4.75km
4.75 - 7.15km
7.15 - 11km
> 11km
