# Supplementary material for: Fine scale human mobility changes within 26 US cities in 2020 in response to the COVID-19 pandemic were associated with distance and income
Source: PLOS Glob Public Health. 2023 Jul 21;3(7):e0002151. doi: 10.1371/journal.pgph.0002151 (PMC10361529; doi:10.1371/journal.pgph.0002151)

S3 Fig. Relationship between relative travel and NPI stringency (left) and case rates (right) at the city level between (a) March 15 – April 12 2020 and (b) June 1 – August 30 2020. NPI stringency was at the state level. Cities with some significant additional NPIs in place (beyond the state-level policies) between June 1 – August 30 are highlighted in red.

(a) There was little association between travel and either NPI stringency or case rates between March 15 – April 12.

(b) When taking into account cities with additional NPIs, there was some evidence of a weak association between NPI stringency and travel between June 1 – August 30. There was little association between travel and case rates over this period.

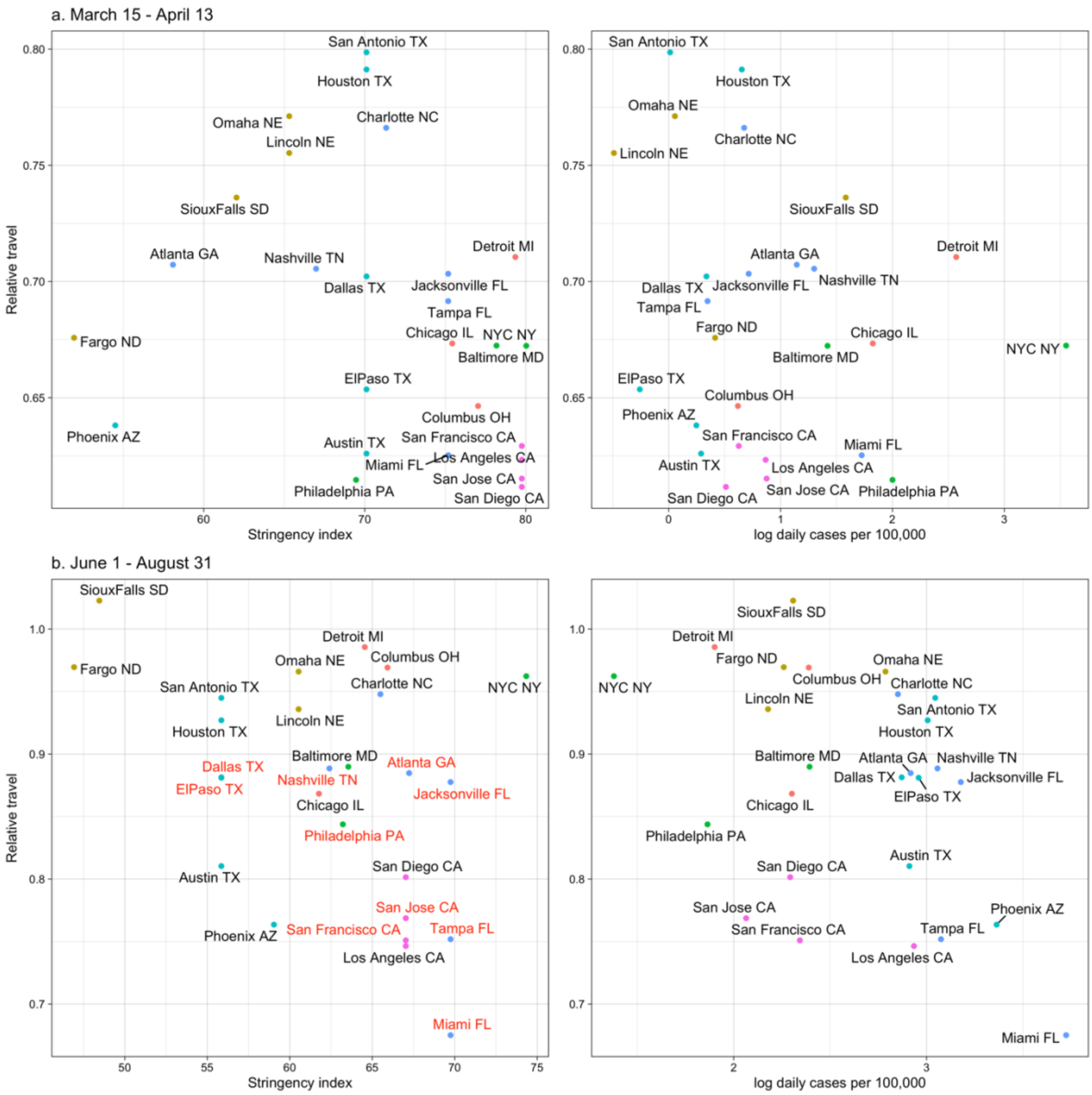

Supplement: S3 Fig — Relationship between relative travel and NPI stringency (left) and case rates (right) at the city level between (a) March 15 –April 12 2020 and (b) June 1 –August 30 2020. NPI stringency was at the state level. Cities with some significant additional NPIs in place (beyond the state-level policies) between June 1 –August 30 are highlighted in red. (a) There was little association between travel and either NPI stringency or case rates between March 15 –April 12. (b) When taking into account cities with additional NPIs, there was some evidence of a weak association between NPI stringency and travel between June 1 –August 30. There was little association between travel and case rates over this period. (PDF) [file pgph.0002151.s012.pdf]
