# Supplementary material for: Fine scale human mobility changes within 26 US cities in 2020 in response to the COVID-19 pandemic were associated with distance and income
Source: PLOS Glob Public Health. 2023 Jul 21;3(7):e0002151. doi: 10.1371/journal.pgph.0002151 (PMC10361529; doi:10.1371/journal.pgph.0002151)

S4 Fig: Daily trips relative to baseline in cities in the South (excluding San Antonio) between June 1 – August 31, with days with data loss removed. Using daily trips reduces the amount of missingness as in the weeks with data loss there was only data loss in some (not all) days. With the exception of Phoenix, where travel decreased during June from above baseline levels to around 90% of baseline, there is little evidence of a decreasing mobility during this time frame. San Antonio is not included as there was a similar amount of data loss in weekly and daily data.

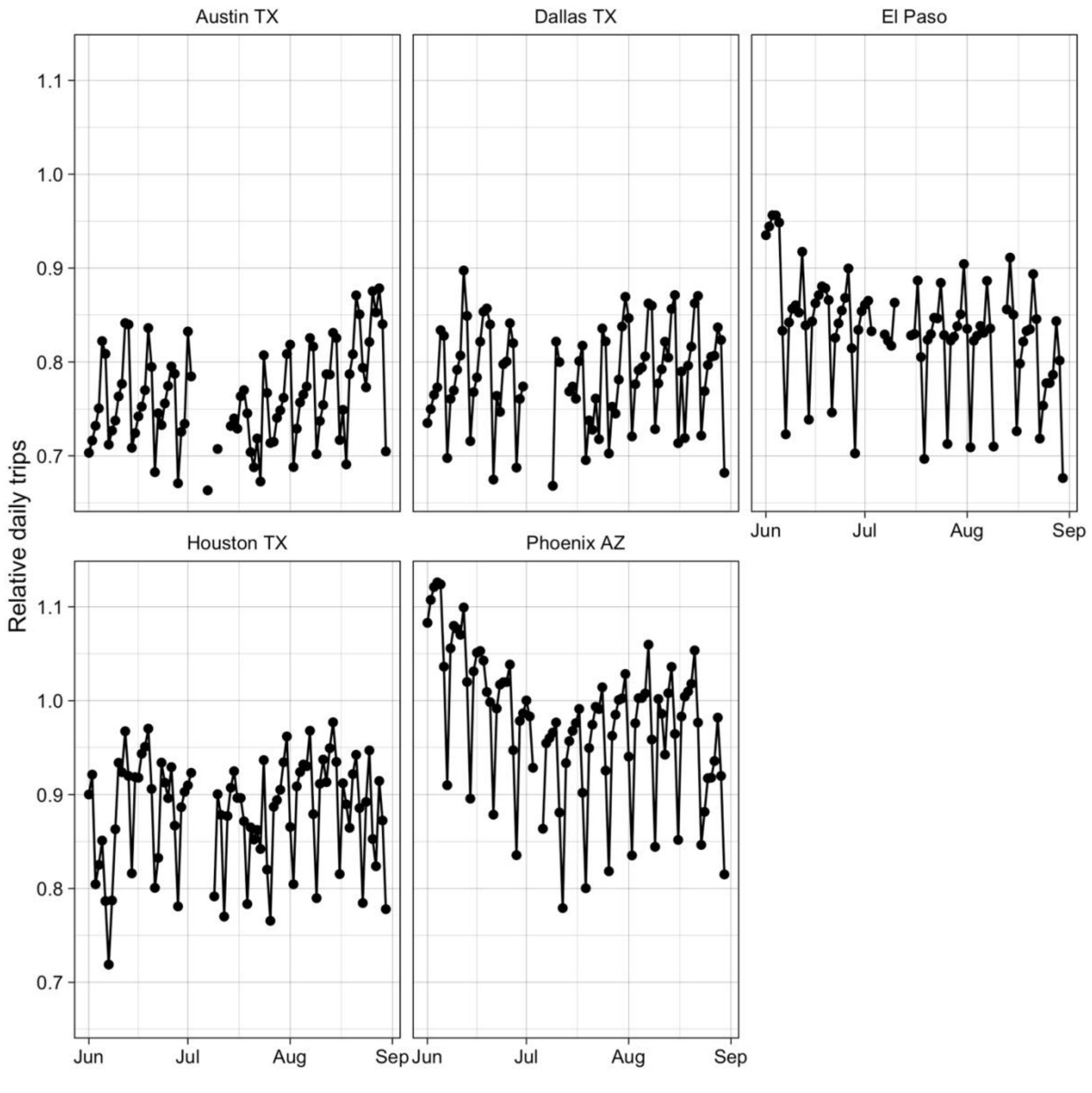

Supplement: S4 Fig — Using daily trips reduces the amount of missingness as in the weeks with data loss there was only data loss in some (not all) days. With the exception of Phoenix, where travel decreased during June from above baseline levels to around 90% of baseline, there is little evidence of a decreasing mobility during this time frame. San Antonio is not included as there was a similar amount of data loss in weekly and daily data. (PDF) [file pgph.0002151.s013.pdf]
