# Supplementary material for: Fine scale human mobility changes within 26 US cities in 2020 in response to the COVID-19 pandemic were associated with distance and income
Source: PLOS Glob Public Health. 2023 Jul 21;3(7):e0002151. doi: 10.1371/journal.pgph.0002151 (PMC10361529; doi:10.1371/journal.pgph.0002151)
Supplement: Attachment — Submitted filename: response_to_reviewers.pdf [file pgph.0002151.s014.pdf]

Dear Dr Ramírez-Soto,

Thank you for the opportunity to address the reviewer comments and to the reviewers for their helpful suggestions. We have revised the manuscript in light of these comments. Please see below for a point-by-point response.

Yours sincerely,

Rohan Arambepola, on behalf of all authors.

<u>Point-by-point response</u>

Reviewer #1: Thank you for the opportunity to review this manuscript. In this paper, Arambepola et al used aggregated US mobile phone operator data to understand the movement patterns across zip codes in 26 cities in the US. Using February 2020 for baseline rates of movement they found that there was overall decrease in mobility early in the pandemic with differences in magnitude varying by geography. Changes in movement across zip codes was associated with trip distance and income level. Age-group associated changes were similar across the different cities. There have been many scientific papers published now on the changes of respiratory virus activity associated with NPI implementation during the pandemic but very few to my knowledge that actually quantify the changes the mobility pattern (one aspect of community level NPI). I believe this manuscript to be of interest to the readers of PLOS Global Public Health. I have some minor considerations below for the authors as they revise their manuscript. Suggest including line numbers for ease of reference during revision.

1. As I started reading the introduction, I found myself asking how the authors defined the scale of mobility in this study. Obviously, mobility can be defined at a number of levels from international travel, interstate and even within neighborhoods and homes. It required that I read the methods before I understood that what this study was about intra-city/zip code travel. The abstract briefly represents zip code-level travel but this is "within the city" travel and not between zip codes of different states. Suggest defining this clearly and early in the title, abstract and introduction.

Response: We have now included that we focused on within-city travel in the title, abstract and introduction.

2. Introduction – This manuscript is packed full of information which I appreciate, however, for the introduction I might suggest hitting the higher level points and reserve more detailed content for the discussion. For example, I think the 3rd paragraph (beginning with "in response to the pandemic,…) could supplement discussions in the discussion rather than being situated in the introduction. Further more, I might suggest that the 4th paragraph be limited to 2 sentences to briefly discuss the objectives and let the rest of the paper go into more methodologic detail. This will help ensure that the messaging is concise and not overly repeated.

Response: Thank you for this helpful suggestion. We have now moved most of the information from the 3rd paragraph of the introduction to the first paragraph of the discussion and significantly simplified the 4th paragraph (now 3rd) of the introduction.

3. The conventional format for many scientific articles has methods prior to presentation of results. In some cases, the order might be enhanced by having results before methods but I think in this case, I would suggest that the methods come before the results. Much of the framework for understanding the results required detailed understanding of the methods and I think the order here of having methods before results is important. Suggest that the authors consider this formatting change.

Response: We agree that including the methods before the results makes sense, especially given the bespoke modelling approaches used. We have made this change.

4. Can the authors describe how the 26 city data were chosen? Why these 26 cities? Or were these the only ones that the mobile providers gave?

Response: We have now added this information to the "Study area" subsection of the methods (lines 6-9, page 4): "These cities were chosen in collaboration with the mobile phone operator based on where sufficient data was available and with the aim of including cities across the country that were, at the time, experiencing different transmission dynamics."

5. Methods – I believe there to be a typo in the age group description in the methods. The age-group 44-54 is missing.

Response: Thank you for pointing this out, we have corrected this on page 4, lines 24-25. "Subscribers were split into three age groups (18-34, 35-54 and 55+ years)..."

6. Methods (Demographic Data) – The sentence beginning "subscribers in the dataset with available income and age data…" is results and not methods.

Response: We have moved this information to the end of the methods section (lines 3-8, page 7).

7. Results – Are the authors able to provide overall description of the dataset? How many mobile providers submitted data? How many unique cell phone users? What was the proportion breakdown by city/state?

Response: We have described the dataset where possible in the first paragraph of the results section (lines 3-8, page 7):

"Subscribers in the dataset with available income and age data over-represented the higher income and older age groups as compared to US census data on the general adult population, see supplementary material section 7 for more details. The share of

subscribers in each city out of all subscribers roughly correlated with the size of each city by population, with the most subscribers in New York (13.3%) and least in Fargo (0.48%). There were at least 5,000 subscribers in each city."

Greater detail is provided in supplementary material sections 7 and 11.

Reviewer #2: Article title reviewed: Fine scale human mobility changes in 26 US cities in 2020 in response to the COVID- 19 pandemic were associated with distance and income.

Main recommendations:
1. Methods section and sub-section Mobile device data: Some weeks in certain cities were excluded from the analysis due to data loss during the collection process (see supplementary material). In general, what was the technique used to process the missing data in this study? That must appear clearly in the article.

Response: Thank you for this suggestion. We have now clarified this on lines 28-31 of page 4: "Some weeks in certain cities were excluded from the analysis due to data loss during the collection process (see supplementary material), which was identified by the mobile phone operator. This data was treated as missing at random. No data imputation was performed."

2. Similarly, the sampling determination method and the data analysis technique must be described and presented clearly as special sub-sections in the Methods section.

Response: Information on the sampling has now been added in the renamed "Sampling and demographic" subsection in the methods section (lines 30-33, page 5): "Data was provided by the mobile phone operator where available but cannot be considered a random sample of the population. Demographic information was used to account for some of the bias introduced by this non-random sampling method…"

While this non-random sampling is not ideal, we believe that by controlling for demographic variables in the regression models we have mitigated much of the bias that could be present in the raw data. We discuss this in the second last paragraph of the discussion (line 43 page 12 - line 14, page 13) and we have quoted the most relevant section below.

The data analysis methods are now described in the methods section in the subsections from line 12, page 5 onwards.

Line 43 page 12 - line 6, page 13:
“Most of the subscribers with demographic data were in the oldest age group in all cities and the higher income groups were also consistently over-represented. As these demographic variables were adjusted for in the regression models, this is unlikely to substantially affect our zip-code level results. However, if older and higher-income individuals were more likely to reduce travel regardless of NPIs in effect then the association between NPIs and rates of travel at the city level may be greater in the general population than we found here. Furthermore, the low numbers of subscribers with data indicating they were in the lowest income group (earning less than $25,000 a year) meant that this group was combined with the next group (between $25,000 and $50,000 a year) in our analysis. Given the evidence of a relationship between low incomes and risk of COVID-19 infection, similar work including higher numbers of lower income subscribers is likely to be valuable.”
